# Eliminating Dog-Mediated Rabies in Sikkim, India: A 10-Year Pathway to Success for the SARAH Program

**DOI:** 10.3389/fvets.2017.00028

**Published:** 2017-03-15

**Authors:** Helen Byrnes, Andrea Britton, Thinlay Bhutia

**Affiliations:** ^1^Vets Beyond Borders, Brisbane, QLD, Australia; ^2^Vets Beyond Borders, Melbourne, VIC, Australia; ^3^SARAH Division, Department of Animal Husbandry, Livestock, Fisheries & Animal Health, Government of Sikkim, Gangtok, India

**Keywords:** SARAH program, Sikkim India, rabies elimination, One Health, mass dog vaccination, dog population management, animal welfare, surveillance

## Abstract

A third of the world rabies burden is in India. The Sikkim Anti-Rabies and Animal Health (SARAH) program is the first state-wide rabies program in India and demonstrates a successful One Health model of dog-mediated rabies elimination. The SARAH program was created in 2006 as a collaboration between the Government of Sikkim and international non-government organizations—Vets Beyond Borders and Fondation Brigitte Bardot. Activities are directed to canine rabies vaccination, humane dog population control, community education, and treatment of sick and injured animals. In 2005, there were 0.74 human rabies deaths per 100,000 (4 deaths) within Sikkim, and from 2006 to 2015, there were no human rabies deaths. In 2016, two human rabies deaths were reported near the West Bengal border region. From 2005 to 2010, the incidence of animal rabies is unknown; from 2010 to 2016, eight cases of animal rabies were reported. Major challenges for the program are continued commitment to rabies control in the face of 0 to low human rabies incidence and the risk of rabies incursions. Effective intersectoral communication between Health, Veterinary, Forestry, and Police officers is essential to enable rapid response to animal bite incidents and possible rabies incursions. An integrated One Health approach needs to be maintained with enhanced active rabies surveillance. Other states must establish similar programs if India is ever to achieve a goal of eliminating dog-mediated human rabies.

## Introduction

**Figure 1:**
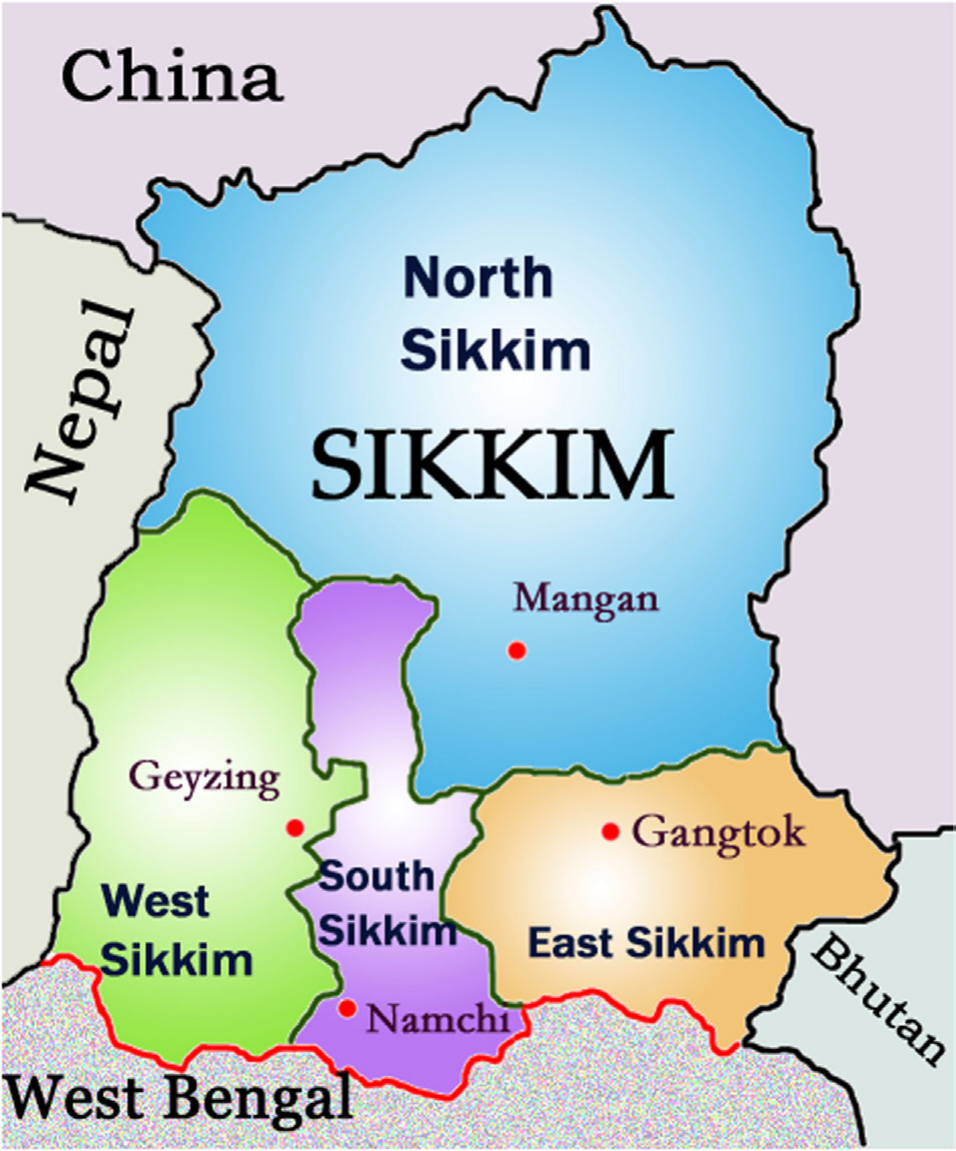
**Map of Sikkim, India—rabies incursions occurred near border of South and West Sikkim and West Bengal border**.

Globally, the incidence of human rabies deaths transmitted from dogs is estimated at 59,000 people, and a third of the world rabies burden is in India (1, 2). In India, there is no national strategy for the elimination of rabies (1), and rabies is not a notifiable disease. Recently, pilot programs for rabies control have commenced in the states of Tamil Nadu and Haryana (3–5), and a number of animal-welfare groups throughout the country include canine rabies vaccination in their activities. The state of Sikkim has implemented a state-wide One Health rabies program since 2006. Sikkim is a small Himalayan state in North East India bordered by Nepal, China, and Bhutan (Figure 1), with a population of 610,000 (2011 census) (6). The core components of the Sikkim Anti-Rabies and Animal Health (SARAH) program (the Program) are canine rabies vaccination, dog population management, and rabies prevention education, which have been shown to control and prevent rabies leading to elimination (7, 8). It also provides health care to street dogs and aims to foster a compassionate attitude toward all animals. The Program enjoys strong community support within Sikkim for its efforts in rabies control and improvements in animal welfare.

The SARAH program was created as a collaboration between the Government of Sikkim, Australian non-government organization (NGO)—Vets Beyond Borders (VBB), and French NGO—Fondation Brigitte Bardot (FBB) in response to the public of Sikkim requesting that mass shooting of street dogs cease and a more humane method of controlling the dogs be implemented. In 2005, human rabies incidence in Sikkim was 0.74 deaths per 100,000 persons, totaling 4 human deaths (Table 1). From 2006, there were no reported cases of human rabies until 2016. Two animal cases were reported in 2010; no further animal cases were reported until 2015. Data for reports of animal rabies cases are poor prior to 2010.

**Table 1:** **Program data on activities and incidence of dog bites and rabies cases, program funding, and volunteers**.

Year	Rabies vaccine doses given	Nos. surgical sterilizations	No. of sick/injured animals treated	No. of public awareness events	Dog bite incidents	Suspect human rabies cases reported	Suspect animal rabies cases reported	Fondation Brigitte Bardot funding (Euro)	Government of Sikkim funding (Euro)	Vets Beyond Borders volunteers (weeks)
2003	0	0	n/a	n/a	n/a	1	n/a	0	0	0
2004	0	0	n/a	n/a	n/a	2	n/a	0	0	0
2005/2006	1,400	830	n/a	n/a	853	4	n/a	12,410	0	81
2006/2007	7,006	4,942	n/a	n/a	n/a	0	n/a	109,000	70,000	103
2007/2008	8,514	5,618	n/a	n/a	n/a	0	0	63,981	57,400	115
2008/2009	7,523	4,364	n/a	n/a	2,320	0	0	70,000	58,212	111
2009/2010	4,941	2,797	n/a	n/a	1,082	0	0	23,000	71,400	79
2010/2011	16,807	3,283	611	n/a	1,334	0	2	21,600	28,000	42
2011/2012	18,611	4,060	1,123	n/a	1,348	0	0	20,500	74,200	47
2012/2013	17,466	2,947	1,581	72	3,315	0	0	14,500	74,900	92
2013/2014	23,669	4,289	2,245	120	n/a	0	0	13,000	61,600	39
2014/2015	23,706	4,300	1,925	86	n/a	0	4	18,000	21,000	39
2015/2016	24,571	5,487	2,304	190	n/a	2	2	22,976	62,300	40

*n/a, not available. Dog bite data provided by Sikkim Department of Health include potential rabies exposure associated with animals such as drinking milk from a cow bitten by a dog or jackal*.

This paper provides a perspective on the implementation of the SARAH program for the control and elimination of dog-mediated human rabies in Sikkim and the benefits and challenges of a One Health approach (7, 9, 10).

## Dog Keeping in Sikkim

Sikkim is largely rural, with 47% of the state under forest cover. Seventy-five percent of the state’s population reside in rural areas. The main urban center is Gangtok (Figure 1) with 100,000 people. Sikkim is a multiethnic state with strong influences of Buddhism (the state religion until Sikkim became an Indian state in 1975) and Nepalese Hindu. The majority of the population is Nepalese (11), and Nepali is the most common language spoken (12).

Dogs in Sikkim are traditionally kept outside as protectors against wildlife intrusion such as Himalayan bear, but also against bad spirits and adverse life events (13, 14). With increased standard of living most village households now keep one or more dogs. In villages, young puppies are often kept inside, and when older are kept outside and handled little. In urban areas, there is an increasing incidence of western-style pet ownership with dogs living inside as members of the family. Workers brought into Sikkim for contract jobs associated with construction of hydroelectric plants usually keep three to four dogs per household. These dogs are kept outside, free roaming and are often difficult to handle. When the contract is finished, the workers move away and usually leave the dogs on the street.

Free roaming dogs are common in Sikkim, and the supervision and responsibility felt for these dogs is on a continuum from nothing to full responsibility. Many family owned dogs are allowed to roam freely. A culture of quasi-ownership has been described in the city of Ranchi, India (15) where people feed roaming dogs but do not take responsibility for vaccination or sterilization, and dog catching with butterfly nets is required for vaccinating these dogs. Over the lifetime of the SARAH program, use of butterfly nets has reduced, and most dogs can now be caught by hand by the SARAH team or community members. While 42% of dogs were reported as stray in Tamil Nadu (5), on average in Sikkim 18% of the total dog population are unsupervised dogs for which no one takes responsibility and which require capture with butterfly nets. In South Sikkim where there are many contract workers, the unsupervised roaming dog population is 27% (Table 2).

**Table 2:** **Estimated number of dogs and canine rabies vaccination in four districts of Sikkim**.

Year	Number dogs vaccinated	Dog population	Number dogs vaccinated	Dog population	Number dogs vaccinated	Dog Population	Number dogs vaccinated	Dog population

	East (% vac East)	East	South (% vac South)	South	North (% vac North)	North	West (% vac West)	West
2011	9,567 (50)	19,000	3,108 (31)	10,000	1,286 (32)	4,000	2,846 (36)	8,000
2012	12,504 (69)	18,200	2,931 (32)	9,200	635 (16)	4,000	2,541 (35)	7,200
2013	12,848 (69)	18,500	2,341 (25)	9,500	598 (15)	4,000	1,679 (22)	7,500
2014	14,458 (76)	19,000	4,932 (49)	10,000	828 (21)	4,000	3,451 (43)	8,000
2015	14,927 (83)	18,000	4,720 (45)	10,500	1,037 (26)	4,000	3,022 (36)	8,500
2016	14,361 (85)	17,000	5,916 (54)	11,000	733 (18)	4,000	3,561 (40)	9,000

*Vaccination coverage is based on dog population size estimates provided by village councils and Department of Animal Husbandry field officers. Feral dogs in forests are excluded from the data estimates*.

There is also a population of feral dogs in the forests in the China border regions, which is likely to be derived from abandoned puppies from army camp dogs. Efforts are made to vaccinate and surgically sterilize these dogs in a trap and release program. The army has permitted access to army camps for the surgical sterilization and rabies vaccination of camp dogs to enable a buffer zone of vaccinated neutered dogs. The army has cooperated with improved garbage control in army camps thus eliminating a food source for feral dogs.

## Community Engagement

Sikkim Anti-Rabies and Animal Health community education on rabies prevention have been designed around core Buddhist and Nepali Hindu religious beliefs including animal sentience, the cherished relationship between people and dogs, and the role of dogs in providing security, and their loyalty and friendship. The Program recognizes that human–animal relationships are “economic, cultural, and emotional in nature” (16, 17) and that dog keeping practices and norms of responsible pet ownership vary in different localities and cultures and can change over time. Animal-welfare lessons were incorporated into early school syllabus in 2009, and further lessons will be incorporated in the 2018 syllabus. The Program has been very careful to address the felt needs of the community to generate community participation.

As trust in the Program has developed, the community is more aware of rabies, and societal norms of animal welfare have changed. Community members will now bring dogs for vaccination and sterilization or describe where they can be found enabling many of the unsupervised dogs to be vaccinated and sterilized. Family planning in women in Sikkim has been actively promoted by the Government (18), and the potential benefits of “family planning” in dogs in reducing the number of unwanted puppies and associated animal-welfare problems were quickly recognized by the community; the community reports fewer dog fights particularly during the breeding season. An increase in dog bite incident reports is seen twice yearly in dog breeding season (March/April and September/October), during the major festival in September/October and following rabies education activities.

Community participation and cooperation is integral to the Program. Key messages to encourage participation are (1) canine rabies vaccination is needed for control of human rabies, (2) surgical desexing will reduce dog roaming and fighting, and hence the risk of rabies, (3) canine rabies vaccination and sterilization are provided free of charge, (4) sterilization will reduce the number of unwanted puppies, and (5) if your dog is unvaccinated and bites a person, you may be held responsible by the Panchayat (local village council) and the affected person for the cost of PEP for the affected person. PEP is available free from public hospitals, but if it is unavailable at the hospital, it must be purchased from a private medical store. In the last 2 years, Panchayats have placed the onus of financial responsibility for PEP on the owners of unvaccinated dogs.

## Stages of Development

The SARAH program has developed over 10 years with capacity building and government commitment from a small NGO-managed program relying substantially on international volunteers to a state-wide government program, providing a One Health model of sustainable dog-mediated rabies elimination.

## Initial Stage

Initially, Program administration, veterinary volunteers, and training were delivered through VBB with a VBB Program manager present in Sikkim and 2–3 international volunteers assisting with the work throughout the year. FBB provided funding on a matching grant arrangement with the Government of Sikkim with the expectation that after 3 years the project would be taken over by the government. Government provided facilities for clinics and public education, accommodation for volunteers, and local staff for the project. A SARAH clinic was established for surgical sterilization, rabies vaccination, and treatment of sick and injured street dogs; mobile units enabled the Program to be extended to rural regions.

Initial and subsequent training activities had a strong emphasis on animal welfare and have been critical for community acceptance of the Program and cost control. Extensive training of local veterinarians in veterinary surgery and medicine, and local staff in animal handling and dog catching occurred through the formal VBB VetTrain© program. Volunteers provided mentoring, on-the-job training, and train-the-trainer programs to local staff. Important elements for community support for the Program were the adoption of humane catching methods, which cause minimal distress to animals and demonstrate a recognition of animal sentience and the significance of dogs in the community, good surgical outcomes with a low rate of surgical complications (<0.003%), rapid return of dogs to their home territory (within 24 h for healthy dogs), and commitment to treat all sick/injured street dogs.

In 2009, the Program became a Division of the Department of Animal Husbandry. VBB volunteers continued to participate, but VBB had a reduced role in Program administration; FBB provided reducing financial support (Table 1).

## Intermediate Stage

Multisectoral cooperation is essential for sustained rabies elimination (19), accordingly, a seminar on rabies and emerging zoonotic diseases was held in 2009. Representatives from the government veterinary and medical fraternity of the State Government were invited; few medical personnel attended. There were two major outcomes from the meeting. The first was the establishment of the Wildlife Conservation and Feral Dog Program to prevent the spread of wildlife rabies into the dog population in Sikkim by creating a buffer zone of rabies vaccinated dogs in the border regions adjacent to China and Nepal.

The second decision was to implement an annual state-wide rabies vaccination campaign each September with World Rabies Day activities incorporated (pet shows, school activities, and media releases). Critically, the implementation of state-wide rabies vaccination extended the Program to the rural regions of Sikkim. Canine rabies vaccine is provided free of charge; annual rabies vaccination of pet dogs is compulsory under state legislation. House-to-house vaccination was needed initially, but central vaccination posts are now feasible in most villages. A catch-vaccinate-release-resight program is undertaken for street dogs with marking of vaccinated dogs with paint. An annual dog census is correlated with village council knowledge of dog numbers facilitating 70% vaccination coverage to be achieved in East Sikkim (20–22). Vaccination coverage by district is shown in Table 2.

A distemper outbreak occurred in 2012 resulting in the death of thousands of dogs and community fear that distemper was caused by rabies vaccine. Extensive community education was undertaken to ensure participation in subsequent rabies vaccination campaigns and encourage owners to vaccinate dogs for distemper.

A One Health Intersectoral Rabies Committee was established in 2012 to transcend sectoral boundaries, comprised of Departments of Health, Animal Husbandry, Forestry, Urban Housing & Development, Police, and Army. The tasks of the committee were to prepare a proposal for rabies to be a notifiable disease in Sikkim, formulate procedures for restrictions of cross-border dog movement, formulate procedures for dog registration, work with the National Centre for Disease Control to establish a State Surveillance Laboratory for rabies control, develop a surveillance system for achieving rabies, and improve garbage control. A major achievement was rabies becoming a notifiable disease in Sikkim in 2014 for animals and humans (23). Garbage management has improved with daily rubbish collection in major towns, and weekly rubbish collection in regional districts.

## Current Stage and One Health Outbreak Responses

Both human and canine rabies were controlled in Sikkim during 2006–2015. Complacency developed about rabies as the perception of disease risk was low. The Health Department stopped stocking PEP and rabies immunoglobulin (RIG), and the need for rabies surveillance and development of laboratory capacity was given low priority. This occurred in the face of complex ecological interactions including wildlife habitat disruption associated with road construction, socioeconomic change, and a migratory workforce located in Sikkim who bring unvaccinated dogs with them and are on the fringe of Sikkim civil society.

In December 2014, two people and a number of dogs were bitten by a jackal in a village close to the West Bengal border. Dog brain samples were sent interstate for testing, but the results were inconclusive. In February 2016, there were reports of a jackal attacking cows near the West Bengal border. Two cows tested positive for rabies (Table 1). Subsequently, two suspect human rabies deaths were reported in a nearby village. One person had been bitten by an unvaccinated pet dog, refused medical treatment, and died. The other person had no history of dog bite and died in hospital. Laboratory confirmation was not undertaken reflecting problems previously identified in effective surveillance of rabies programs (24): inadequate training in sample collection, difficulties in getting samples to a diagnostic laboratory from Sikkim, and family reluctance to allow postmortem diagnosis.

## Discussion

### Vaccination and Dog Population Control

Rabies vaccination of dogs is the cornerstone of rabies control (25, 26). The logistics of a state-wide vaccination campaign in the Himalayas are difficult. Cooperation of villagers, Panchayats, and Department of Animal Husbandry field officers is essential. Human-mediated dog movement and gaps in coverage are problems in effective rabies programs (27). Recent rabies incursions in South Sikkim occurred in areas where there is human-mediated dog movement, where they have been gaps in vaccine coverage (Table 2), and which is adjacent to West Bengal with no rabies program. A Program team is now permanently located in South Sikkim to improve vaccination coverage and dog population management.

Dog population management is important for the Program goals of improved animal welfare (28, 29) and rabies control; the numbers of dogs sterilized annually are approximately 20% of the dogs vaccinated in Sikkim each year (Table 1). It is also important because it addresses community concerns about dog fighting and nuisance, and unwanted puppies. Improved animal welfare supports a more stable dog population. Community assistance with dog catching enables a minimal although highly skilled dog catching team. The aides who assist with surgery are also the dog-catching team. This has enabled control of a major cost, for the size of the dog catching team can be a significant cost in a dog population management program (3).

### Intersectoral Coordination, Community Engagement, and Animal Welfare

Intersectoral coordination and communication, essential for rabies control (10, 19), is an ongoing challenge. The establishment of a One Health Intersectoral Committee provided the authority for SARAH to seek cooperation at district and village level. The perceived success in controlling rabies and dog population management together with wide community support facilitated cooperation at all levels, although an unanticipated outcome was the Department of Health’s interim decision to stop stocking PEP and RIG.

Education on animal welfare and the obligation to care and value dogs has been associated with increased community participation and support for the Program. There is a temptation to assume that the animal welfare emphasis of the Program will only work in Sikkim where animal sentience is accepted and the complex relationship between people and dogs is acknowledged in festivals such as Tihar (Deepawali). It has been suggested that the important role of dogs in Hinduism may be an impediment for successful program adoption (30), but in Sikkim, it has facilitated program adoption. An increase in empathy and improved attitudes to animals has been shown to increase empathy to humans and facilitate prosocial behavior (31–36), which may in turn motivate health behaviors including participation in vaccination campaigns (28). A critical feature in the SARAH program is the recognition of the significance of human–animal relations and culturally appropriate framing of community education messages.

### Challenges Facing the SARAH Program

Recent suspect rabies cases highlighted the need for formal and regular intersectoral communication at community level, the need for improved epidemiological data, for enhanced active surveillance related to animal bites, training for appropriate medical response to suspect rabies dog bite, the logistical difficulties in getting both human and animal samples from Sikkim to a laboratory for confirmation of rabies, and the ongoing risk of rabies incursion and sylvatic rabies.

The seasonal pattern of the recent rabies outbreaks occurring at the border during winter when food sources are scarce suggests rabies incursions into Sikkim, rather than ongoing circulation of rabies within Sikkim. An effective surveillance system with tracing back of suspect animals is needed to confirm this hypothesis (37, 38). Increasing human wildlife conflict in Sikkim (6) may increase the risk of rabies transmission. If rabies were controlled in domestic dogs in surrounding areas, it is not known if jackal could sustain the circulation of rabies although Lembo (20) concluded that dogs were the only species essential for rabies persistence in the Serengeti. There are clear limitations in the accuracy of vaccination coverage estimates based on dog population size estimates provided by village council knowledge. However, the data suggest that vaccination coverages of 70% are likely to be feasible in Sikkim, as shown by consistently high estimates achieved in East Sikkim.

The SARAH program is a State Government supported program and lacks the international resources available to national programs. Effort is being directed at establishing low cost enhanced active rabies surveillance (37). The Rapid Test (BioNote) is used in the field when available. Discussions are being held with counterparts in West Bengal for extension of the rabies program into West Bengal, but resources are limited for both parties.

## Conclusion

The Sikkim Government, together with SARAH partner NGO—VBB and FBB, has made a considerable investment to eliminate dog-mediated rabies. The recent re-emergence of rabies in Sikkim highlights the imperative of an integrated One Health approach to increase the sensitivity of rabies surveillance and to ensure interruption of rabies transmission. The SARAH program is a model of rabies control in a predominantly rural environment with limited resources. It is also an example of the challenges encountered in maintaining rabies control in a landlocked state. Other Indian states must establish similar programs if India is ever to achieve a goal of eliminating dog-mediated human rabies.

## Author Contributions

HB has been involved in the SARAH program since 2006. HB and AB researched, designed, and wrote this paper. TB has been local coordinator for the program since inception and provided expert and local knowledge about the paper subject.

## Conflict of Interest Statement

The authors declare that the research was conducted in the absence of any commercial or financial relationships that could be construed as a potential conflict of interest.
